# Facilitators and Barriers to the Development of Health Literacy Capacities Over Time for Self-Management

**DOI:** 10.3928/24748307-20200221-01

**Published:** 2020-05-08

**Authors:** Verna B. McKenna, Jane Sixsmith, Margaret Barry

## Abstract

**Background:**

Health literacy is a dynamic construct that is content and context specific. An understanding of the facilitators and barriers involved in the development of health literacy over time can provide important insights for the health care providers (HCP) in supporting patients with chronic illness.

**Objective:**

The study was conducted to expand an understanding of how health literacy development can be supported through exploration of the main facilitators and barriers in the process.

**Methods:**

This study used a longitudinal qualitative study design involving repeat interviews at three separate time points over a 12-month period. A purposive sample of 26 participants attending a structured cardiovascular disease risk-reduction program participated in the study, 17 of whom completed all three interviews. The European Health Literacy Survey measure was used to determine health literacy levels at the beginning and end of the 12-month period. Employing qualitative thematic analysis and a longitudinal-specific question framework, a trajectory approach was applied to explore individual cases longitudinally.

**Key Results:**

Facilitators and barriers to health literacy capacity development were identified. Participants demonstrated increased perceptions of having control and being empowered over time. However, this was also found to be affected by external life events. Study participants were also found to be embedding health knowledge, motivation, and behaviors over time within the everyday contexts of their lives. The relationship with the HCP permeated all aspects of health literacy capacity development, including aspects of treatment decision-making. Participants identified the need for psychological supports and the increased importance of looking after their mental health.

**Conclusions:**

Positive developments in health literacy capacities are important for the self-management of illness. Longitudinal findings underscore the importance of the HCP in supporting the development of health literacy capacities over time. These findings lend support to the need to integrate health literacy into medical and other HCP curricula to raise awareness of the concept of health literacy. **[*HLRP: Health Literacy Research and Practice*. 2020;4(2):e104–e118.]**

**Plain Language Summary:**

Using a longitudinal qualitative study design, this study proposes that health literacy capacities develop over time and that the health care provider (HCP) plays a central role in this process. Findings from this study support the need to embed health literacy training into medical and other applied HCP curricula to raise awareness of the concept of health literacy.

Health literacy is content and context specific and concerns the capacities of people to meet the complex demands of health in modern society (Nutbeam, 2015; Sørensen et al., 2015). It is closely linked to the main tenets of health promotion, whereby it is viewed as a personal and population asset for achieving greater autonomy and control over health decision-making (Nutbeam, 2015; Nutbeam, McGill, & Premkumar, 2018). Health literacy is an important factor in the maintenance and improvement of health (Berkman, Sheridan, Donahue, Halpern, & Crotty, 2011; DeWalt, Berkman, Sheridan, Lohr, & Pignone, 2004) and is also considered to be a crucial component in the self-management of illness (Coulter & Ellins, 2007; Levin-Zamir & Peterburg, 2001; Lloyd, Ammary, Epstein, Johnson, & Rhee, 2006; Sorensen et al., 2015).

Limited health literacy is an invisible barrier to health care delivery and a barrier to effective patient care (Magnani et al. 2018; Seurer & Vogt, 2013). Research has consistently found that people with low health literacy experience poorer health outcomes across a wide range of areas and poorer use of health services. A systematic review by Berkman et al. (2011) identified this association for use of preventive services, self-management of illness such as adherence to medication, and increased admission to the hospital and longer hospital stays. The Health Literacy Pathway model developed by Edwards, Wood, Davies, and Edwards (2012) describes developments in health literacy for people but is overly focused on responding to ill health rather than acknowledging social determinants of health and the role of health promotion (Guzys, Kenny, Dickson-Smith, & Threlkeld, 2015). A person's health literacy is dependent on the relationship between individual capacities, the health care system, and broader society. Barriers to use of health literacy capacities include socioeconomic circumstances, social support, as well as the nature of the health care setting (Jordan, Buchbinder, & Osborne, 2010). People who have developed higher levels of health literacy will have skills and capabilities that enable them to engage in a range of health-enhancing actions (IUHPE, 2018). Edwards at al. (2012) identified further barriers in the use of health literacy skills in terms of personal, emotional, and health professional barriers.

Research has indicated that improving self-efficacy levels in patients can result in increased confidence in making health behavior changes, which is fundamental to self-management (Bodenheimer, Lorig, Holman, & Grumbach 2002). Models describing self-management behaviors highlight the three patient attributes of knowledge, self-efficacy, and beliefs that, when combined, are important for effective self-management (Bodenheimer et al., 2002; Lawn & Schoo, 2010; Wingham, Harding, Britten & Dalal, 2014). These also correspond to the mediating factors identified by Paasche-Orlow and Wolf (2007) in their model of possible causal pathways between health literacy and health outcomes. A review of the impact of health literacy on self-management skills suggests a link between those skills and self-management skills but calls for an increased emphasis on intervention studies for further examination (Mackey, Doody, Werner, & Fullen, 2016).

Self-management strategies can result in improved health outcomes for those with chronic diseases (Ruiz, Brady, Glasgow, Birkel & Spafford, 2014), and health literacy has been identified as a potential facilitator or barrier to improved health outcomes (Mackey et al., 2016). Low health literacy is associated with poorer self-management skills (Mbaezue et al., 2010; Naik, Street, Castillo, & Abraham, 2011; Nutbeam, 2008).

Health literacy is crucial to enable people to manage their health. Much of the self-management of chronic diseases is performed by patients outside of the medical or health care setting. Often this care is complex. Medication adherence frequently requires understanding complex scheduling and dosing details, as well as information relating to dietary choices and timing and appropriate vigilance about symptoms and side effects (Boren, 2009; Magnani et al., 2018).

Nutbeam's (2000) model of health literacy, involving functional, interactive, and critical levels, can be applied to self-management (Heijmans, Waverijn, Rademakers, van der Vaart, & Rijken, 2015). Health literacy is conceptualized in this model as involving skills at various levels that have an ascending order of complexity and can gradually lead to greater personal autonomy and empowerment (Smith, Nutbeam, & McCaffery, 2013). A number of studies have indicated that interactive and critical health literacy are stronger predictors for successful self-management than functional health literacy (Heijmans et al., 2015; Lai, Ishikawa, Kiuchi, Mooppil, & Griva, 2013; van der Heide, Heijmans, Schuit, Uiters, & Rademakers, 2015), suggesting that more complex skills are involved.

Although there has been a recent proliferation in research studies in health literacy (Nutbeam, Levin-Zamir, & Rowlands, 2018), increased insight is needed into how the development of these complex skills can be facilitated. A qualitative longitudinal study design allows for an increased understanding of health literacy challenges experienced in the management of health and illness over a period of time and of the strategies people might use to address such challenges. This is an important consideration for health care providers, especially in relation to self-management programs, to highlight specific areas where more supports may be necessary to facilitate the shift from functional to critical health literacy capacity development. Increasing rates of chronic illness worldwide will place increasing demands on health systems (Busse, Blümel, Scheller-Kreinsen, & Zentner, 2010). One way to ameliorate the effects of this is to engage patients in more effective self-management (Heneghan et al., 2009). Health literacy is central to this approach (World Health Organization, 2016). However, having an understanding of how health literacy developments can be supported is crucial. This is the focus of this study, which offers the potential of identifying important levers and vulnerable points in the development of health literacy where more intensive supports may be needed. The study seeks to expand the understanding of how health literacy development can be supported through addressing the following research questions: (1) does health literacy develop over time in the context of prevention and health promotion interventions? and (2) what are the main barriers and facilitators to HL development?

This study employs a qualitative methodology incorporating the European Health Literacy Survey conceptual model to explore people's experiences. This is a recently developed comprehensive model of health literacy that emphasizes the capacities necessary to be considered health literate and to make decisions about health access, understanding, appraisal, and application that can be linked to functional, interactive, and critical levels of health literacy (Sørensen, et al., 2012).

## Methods

### Study Design

A longitudinal qualitative design (**Table 1**) was deemed most appropriate for this study, as the main aim was to generate data and an in-depth understanding of a person's perspectives and experiences of health literacy capacities and how and why these might change over time (Corden & Millar, 2007). The number and frequency of serial interviews that compose a longitudinal study is dependent on how a given research problem is posed and how it will vary from study to study. In terms of deciding on how much time should pass before successive interviews are conducted, the answer is the amount of time sufficient to examine relevant change from one point to another (Hermanowicz, 2013; Saldana, 2002). In this study, the longitudinal element covered 12 months, which is an appropriate timeframe in longitudinal qualitative research (LQR) (Holland, Thomson, & Henderson, 2006). The timeframe was also deemed apt to discern changes in health literacy capacities and dovetailed with the timeline of the risk-reduction program that participants were attending. Findings directly relevant to time points 1 (T1) and 2 (T2) have previously been published (McKenna, Sixsmith, & Barry, 2017, 2018). This article presents the overall longitudinal findings for 17 participants from T1 to T3 and explores their development of health literacy capacities over a 12-month period. The aim of this study is to understand people's experiences over time in using health literacy capacities, including facilitators and barriers, to manage their health and illness. Specifically, this study adopts a trajectory approach to describe how experiences change over time (Grossoehme & Lipstein, 2016).

## Participants

The sample frame for this study was people attending a community-based, structured cardiovascular disease (CVD) risk-reduction program. Purposeful sampling was used to identify people with a range of risk factors for CVD, as well as those with established disease, to obtain their views and experiences (Creswell, 2013). The program was used as a “vehicle” to engage people as they managed their health and illness over a 12-month period. There were 26 people interviewed at T1, 19 at T2, and 17 at T3 due to attrition of seven participants over the duration of the study.

Initial recruitment took place in conjunction with the program nurse, who identified people who were cognitively able to participate and had an understanding of the English language. Recruitment for T1 took place between February and December 2014, and the sample was observed over a 12-month period with final T3 interviews taking place in January 2015.

### Profile of Study Participants

All of the participants (*n* = 17) had completed a 12-week CVD risk reduction program and were referred through various pathways including general practice and hospital departments such as cardiology, stroke, and endocrinology. Participant characteristics, including health literacy levels recorded at T1 and T3, are summarized in **Table 2**.

## Data Collection Procedures

### Interviews

Semi-structured interview guides were used to explore the development of HL and to identify changes in knowledge, attitudes, and experiences over time. The development of the interview guide was informed by Sørensen et al.'s (2012) conceptual model of health literacy to explore all the capacities associated with health literacy. Interview questions across T1 to T3 focused on the areas of accessing, understanding, appraising, and applying health information, and transcript data were initially categorized within these areas. In addition, questions were also included to explore further issues that had been identified in T1 and T2 of the research (e.g., concerns about upcoming treatment decisions). The interview schedule was piloted prior to commencement of data collection with a small number of people attending the structured program. All interviews took place at the community-based program building and were conducted by the first author (V.M.K.). Retention issues and attrition of participants is common in qualitative longitudinal studies (Hermanowicz, 2013; Murray et al., 2009). In this study, attrition was attributed to a combination of issues including a limited engagement with the risk-reduction program and illness factors that prevented program completion.

### Data Analysis

All interviews were audio recorded digitally, transcribed verbatim, and analyzed using thematic analysis (Braun & Clarke, 2006), which was facilitated through the use of N-Vivo (version 10) qualitative software. Qualitative validation criteria were applied in this study in line with established guidelines (Creswell & Miller, 2000; Guba, 1981; Maxwell, 1992) as shown in **Table 3**.

The study used a hybrid approach of inductive and deductive coding and theme development, employing a thematic analysis methodology as advocated by Braun and Clarke (2006). Longitudinal analysis included summarizing and comparing the data both cross-sectionally and longitudinally (Thomson & Holland, 2003). A matrix format (Grossoehme & Lipstein, 2016; Miles, Huberman, & Saldana, 2014) using Microsoft Excel 2013 was employed to facilitate the ordering and summarizing of data for each participant across T1 to T3. Trajectory analysis, which focuses on changes over time for a person or small group of people, was used to meet the aim of the current study (Grossoehme & Lipstein, 2016). Sample matrices and an overview of the process are available in **Figure A**.

Longitudinal question frameworks (Lewis, 2007; Saldana, 2002, 2003) (**Table A**) were used to ensure that the analysis captured the process of development and changes rather than presenting cross-sectional findings only (Calman, Brunton, & Molassiotis, 2013; Saldana, 2003). By linking back to the previous data set, it was also possible to determine what changes or developments had occurred in terms of health literacy capacities. Preliminary analysis took place between interviews at T1 and T2 and at T2 and T3 to allow reflexivity on the part of the researcher (Carduff, Murray, & Kendall, 2015) as well as to focus on process and changes rather than on snapshots (Calman et al., 2013). This preliminary analysis allowed the researchers to identify key issues that could then be returned to for further exploration in subsequent interviews.

In this study, data saturation was reached in terms of inductive thematic saturation (analysis focus) and data saturation (data collection focus) as outlined in models of saturation put forward by Saunders et al. (2018). In addition, phases 3 to 5 of Braun and Clarke's (2006) methodology for thematic analysis (searching for, reviewing, defining, and naming of themes) were applied, involving a process of checking for theme saturation to ensure that all data fit the themes and that no new themes are identified.

### Ethical Considerations

The study was independently reviewed and approved by the Research Ethics Committee, National University of Ireland, Galway in May 2013. All participants were provided with written and oral details of study participation and provided with written informed consent to participate in the study. Emphasis was placed on the voluntary nature of study participation, with the removal of all identifiers and assurance that all information would be anonymized. Due to the nature of longitudinal research, consent should be viewed as a process rather than an initial act (France, Bendelow, & Williams, 2000) In this study, consent was requested from each participant at each time point. The Participant Information Sheet specifically set out that all participation was voluntary and that participants were free to opt out of the study at any point.

## Results

Building on findings from T2, the overall longitudinal findings indicate that developments occurred across the different levels of health literacy (functional, interactive, and critical). However, there were individual variations in these developments contingent on personal experiences and contexts.

Four themes were identified from this longitudinal analysis, and together with sub-themes and categories they are presented in **Table 4**. Barriers and facilitators in the development of health literacy capacity were evident within all four themes and are described below. Quotation labels are numbered by participant (P) and also denote gender (M, male; F, female) and health literacy level at T3 (A, adequate; L, limited).

## Gaining Control and Becoming Empowered

Analysis of data across T1 to T3 indicated that, overall, participants gained a greater sense of control over their health and illness over the 12-month time period, which facilitated the development of health literacy capacities. However, the potential for gaining increased confidence, and control could be affected by the experience of adverse circumstances in terms of illness or other demands placed on the person. External life events (such as taking care of older relatives) affected their psychological ability to effectively use and develop their health literacy capacities, and in this regard acted as a barrier.

### Self-Efficacy

Many participants experienced positive growth in confidence associated with changes in health practices that were sustained over time. Being able to see real change, such as improved weight, contributed to self-efficacy and the understanding of personal ability to exert control over one's life circumstances and health issues.

I thought I never could [lose weight], I thought there was nothing I could do about it and yet there was. I think I'm more confident now in knowing that I can do things too, that if I wanted to change something I can

(PF23L).

### Psychological Impacts of External Events

Changes in life and health circumstances of the participants themselves or of close family members had an impact on their perceived control and confidence, either positively or negatively. For some, external life events acted as barriers to their ability to engage with health issues.

I don't feel I have any control at the moment. Sometimes I don't leave my mum's house until late in the night, I'm too tired. I suppose I'm emotionally drained, so it's difficult. So that impacts on my life a lot

(PF8L).

Over the 12-month period, participants moved from focusing mainly on physical aspects of their health to identifying the importance of looking after their own mental health and linking its relevance to sustaining physical health and managing lifestyle plans. One participant identified the practical and emotional strains of caring for older family members, combined with upset over a daughter's recent emigration and linked these events to her engagement in comfort eating:

I eat when I'm emotionally not in a good place. I'm always thinking ‘Oh God I shouldn't be doing this’ and ‘I'm mad with myself that I put on weight since October’ – that I didn't kind of pre-empt having all this additional kind of stress would cause me to eat more

(PF8L).

### Looking After Self

Over the 12-month period, developments were apparent in participants' abilities to reflect back on and re-evaluate events, including the role of stress and the impact of grief. One participant reflected on how ongoing stress had negatively affected him and the adverse effect it has on his blood pressure. He reflected on how this was intensified due to living on his own and being unemployed. However, his more recent engagement with employment has had a positive impact on his mental health:

Because now I know, I suppose I went so far down and so deep that I didn't know what way to fight back. And now I'm gone to the stage that – I will never go back there again

(PM13A).

Having coped with illness in one's self and others, participants start to see the importance of looking after themselves:

Well in looking after myself, concentrating on what I want to do, and doing the exercises for me. Thinking about myself more, not worrying about my children or grandchildren, they're going to be fine. This is my time

(PF15LA).

### Need for Psychological Supports

Together with an increased awareness of the significance of mental health, participants also spoke about the importance of, and need for, psychological supports at certain times during illness management. However, it was also acknowledged that this could be difficult to access in terms of costs and knowing how or where to access services. Participants recognized the importance of being able to talk through psychological aspects such as coping and fears and anxiety about health problems. There is now a realization that mental health matters have to be addressed, and mental health looked after, to have the ability to take care of the physical aspects of health.

One participant described how she had felt when the support of the program ended and reflected how appointments with a general practitioner (GP) do not allow time for talking through emotional concerns:

I think it was something I'd been holding in for, since I was diagnosed nearly. And it was good to actually have a heart to heart talk with somebody because doctors don't have any time really. I think I've had excellent care, I'm blessed with medical care. But the talking bit is missing

(PF21L).

Coping with a new illness requires making adjustments, facing limitations, and dealing with new challenges. New illness requires re-engagement with new information and new ways of using health literacy capacities, but this can be obstructed by fears and anxieties. Empowerment and increased control experienced over time could be diminished by the onset of a new illness due to the fears and anxieties of coping with a new situation.

One participant who had experienced serious illness since T2 reflected on the mental challenges after hospital discharge and adjusting to new limitations:

I could see all the negative things really, and if I got a twinge, or as I said, if you coughed twice, you were thinking ‘oh God is this coming back again?

(PF5L).

### Embedding Knowledge, Health Practices, and Motivation

It is clear that study participants have continued to embed health practices up to 9 months after program completion. Many of these practices center on diet and exercise and there is an increased awareness of the importance of the combined effect of the two practices together to obtain the greatest benefits. Participants were also surprised at how manageable it was to make dietary changes seeing that it really required only small changes over a period of time and the effect on self-efficacy and motivation. This engagement with health practices and knowledge facilitated the development of HL capacities over time, including developments in motivation.

### Accessing and Using Information

Participants continued to develop knowledge about health and illness issues over the duration of the study. In some instances, apprehensiveness at T1 about concerns had been replaced with confidence in being able to access and understand new levels of knowledge over the 12-month period of the study.

Do you know because I knew more and I read more about it, I just flew through everything belonging to it, you know that kind of way

(PF1A).

In the case of a new onset of illness, participants had to navigate a different illness context using health literacy capacities. Participants indicated a confident approach to gaining information and assistance as needed. One participant had drawn on a number of different resources to access information for her oxygen treatment plan:

If I saw anything now in a paper or a magazine, straight away I would read it and keep it. Even like about the oxygen because I knew nothing really about oxygen, or lacking in oxygen but I found the company that supplies the oxygen now, they would be very good

(PF5L).

However, some participants still struggled to fully access information and to understand all aspects of their condition:

I'm still not clear on the type of cardiomyopathy I have and even the night I went to [out of hours doctor service], the doctor said ‘so is it the genetic, something?’ and I didn't know. I still don't know that. I haven't got a clear answer from [Dr] really or maybe he has and I haven't taken it in, because one thing I've learned is that when you're sick you cannot think. Or my memory went completely and my taking in of information was terrible

(P21L).

### Environment

Participants have sustained an awareness of the broader determinants of health, evident in the importance placed on the living environment, which can impact on health practices and health outcomes. Participants continued to reflect on their environments in terms of local community, living space, and availability of facilities to engage in health pursuits (walking, swimming, gym). The environment and access to facilities also impacted on motivation to engage in health practices. Rural environments are seen on the one hand as a peaceful and a positive place for raising children that contribute to a natural healthy way of life, but they also present challenges in terms of transport and facilities to engage with health practices. The availability of local facilities in more urban areas makes healthy pursuits more feasible.

The environment – I've mentioned to you before what would make a big difference is paths on roads, now they are doing a whole major roadworks approaching the village, and there is talk that there'll be a cycle lane and a path – so that would be quite nice

(PF8L).

However, the experience of antisocial behavior in an urban neighborhood can impact on the ability to get out and can also negatively impact on mental health.

And there's the little thugs as well. And the guards are always up and down to them. ...and all the damage is done, they're breaking trees and everything up there – throw stones at windows and doors. I'm telling you, you're a prisoner in your own house at night, because you can't go out, because they're hanging around the area

(PM10L).

### Food Literacy

Sustaining practices around diet go beyond an understanding of what type of foods one should buy and eat. Participants are now also identifying the relevance of cultural, social, and family practices and how these can impact on food choices and being able to maintain healthier practices. For example, in cutting out sweet foods and baked goods, there were concerns around not having anything for visitors. There was also recognition that the marketing and proliferation of cheap offers on biscuits, cakes and sweets can make it difficult to stick to healthy eating plans:

We don't have it around as much, we don't have the chocolate or crisps. Oh yeah and every one of them [grandchildren] came into the house, they used to say ‘it's not fun here anymore’, even my daughters, there was no, when there was no biscuits for the tea and things like that, they all kind of got used to it

(PF23L).

Family practices can also impact on food practices. One participant describes the challenges of adhering to low-cholesterol diet when sharing meals with family:

Well, left to my own devices now, so when I'm on my own I will do whatever I have to do in the morning, and then I might have a salad at lunch time. But if my wife or my daughter or my son are in the house, lunch time could be anything, do you know, everyone is coming in at different times, and next thing there is soup and sandwiches, or different things being eaten like, and you participate. Like other people in the house don't have cholesterol problems, so they can get away with eating different things, you know. So from that point of view like I have to kind of cater for myself and that

(PM16L).

Being able to read and understand food labels also impacts on food buying choices. There is also an increased awareness on how advertising can use language that is confusing to the consumer:

The advertising, because you get these, all these ninety percent fat free and you say ‘that's fantastic’ but it's an outrageous amount of fat in a small little container of yoghurt or whatever

(PF4A).

## Dynamics of Relationship and Support of Health Care Providers

The impact of the relationship with the health care provider (HCP) on development and use of health literacy capacities is evident in this study. Positive interactions facilitated engagement and development of HL capacities, whereas more negative interactions acted as a barrier. These interactions are particularly important for accessing and appraising health information. The HCP may facilitate or impede the process depending on the nature of the relationship and the quality of rapport, communication, and support in that relationship. The GP remains the main source and most trusted source of information for many and also plays an appraisal role when problems are discussed. However, negative experiences were also highlighted, including concerns about missed and delayed diagnoses and difficulties in seeking referrals. Participants are generally reluctant to leave a GP and to seek out another practice. In some cases, the overall perception of a good relationship with the GP supplants concerns about the service being provided.

### Communication, Rapport, Trust, and Approachability

Positive HCP experiences centered on having a good rapport, clear communication, and a non-paternalistic approach. One area of concern was difficulties in securing a referral.

One participant identified a strategy of using the locum to get a referral as she feels her own GP is reluctant to make referrals:

But sometimes I think he's not great at it if you feel you want to be referred to someone – he doesn't do that so much. But what I've done–I discovered when the locum was in, so I went back to her. Immediately she wrote a referral letter. So that's my way. I'll deal with her

(PF8L).

Having a good rapport is of a paramount importance even where there are concerns that an illness was not diagnosed in a timely way:

Like on the one hand – maybe the ovarian cancer thing I kind of think, why was it missed? on the other hand I have a good relationship with him, and I think that is important. You know he kind of understands me after all these years

(PF8L).

During the course of the study, participants also engaged with HCPs on the structured program. Participants reflected on the positive aspects of access to a multidisciplinary team, the approachability of staff, as well as the encouragement and non-paternalistic approach offered. This was linked to enhancing motivation for the participants.

I think between the whole lot, and the fact that we got the exercise, the dietician and the nurse and we got on so well with them. They seemed to be so interested in us that I felt that I didn't want to let them down either

(PF23L).

## Treatment Decision-Making

Health literacy capacities are needed to engage with treatment decision-making and to make relevant decisions. Where access to relevant information or understanding is limited or conflicting information is provided, barriers to participation with treatment decision-making can occur.

### Managing and Challenging Side Effects

Participants were knowledgeable about the side effects of medications they were taking and were engaged in seeking solutions to address the side effects. Different strategies were used for this which seemed to be impacted by the relationship and level of communication with the HCP. The majority sought advice from HCPs, but some did not. Participants were proactive in raising issues regarding medication side effects with medical teams and requesting changes. As in previous interviews, some participants were concerned about the side effects associated with the use of medications to lower cholesterol. Some of these concerns were linked to media reports about cholesterol-lowering drugs.

Conflicting advice regarding medication use is confusing and upsetting for patients. One participant had experienced severe dizziness on a certain drug. When he mentioned this to his HCP, it was agreed that this was a typical side effect from long-term use and that another medication could be prescribed instead; however, another consultant told him to continue taking the medication.

Sometimes fears about medication side effects were based on reading up on the side effects rather than the actual experience of them. Being able to discuss concerns with a doctor was found to be helpful.

And I went back to say I want to get off of these because I don't like what I seen about the side effects. He said “look we'll try and cut down” because he said “if you go off them now straight away the pain might get worse

(PM10L).

### Decisions About Treatments

For some participants, fears about treatment and their understanding of what it entailed could impact on delays in seeking treatment. In some cases, this was based on not having a clear understanding of what a treatment procedure actually involved. One participant had delayed taking up testing for sleep apnea due to her concerns about what the treatment actually involved.

“I had this imagination in my head of what I'd seen on the television, of you being inside in a room all wired up and they're all sitting outside watching. It turned out like to be completely different”

(PF5L).

## Discussion

This study set out to examine developments in health literacy over time and to identify the facilitators and barriers in that process. Study findings support the conceptualization of health literacy as an asset (Martensson & Hensing, 2012; Nutbeam, 2008, 2015; Pleasant & Kuruvilla, 2008). Developments across the three levels of health literacy (functional, interactive, critical) were also apparent, simultaneous with a progression in personal empowerment as advocated by Nutbeam (2015). Such progression is also contingent on a person's self-efficacy, which was also evident in the current study. The study findings also showed that developments in interactive health literacy were apparent in interactions with HCPs together with the appraisal and discussion of medication side effects and treatment options. The identification of environmental facilitators and barriers to health promotion and healthy living, as well as a shift toward a greater focus on addressing psychological issues, is indicative of critical health literacy (Chinn, 2011).

This study was underpinned by the health literacy survey model empirically validated by Sørensen et al. (2012) whereby health literacy is recognized as a process involving the consecutive competencies of accessing, understanding, appraising, and applying health information, which also links health literacy to its antecedents and consequences (Sørensen et al., 2012). According to the model, application of the competencies provides people with the ability to take control over their health by overcoming personal, social, structural, and environmental barriers to health. In this study, participants were identifying barriers and moving toward addressing them, such as the need for better walking facilities and the need to mind their mental health. Study findings also support the contention that health literacy is a dynamic construct and that the skills and competencies of health literacy develop over the life course as contextual demands change over time (Levin-Zamir, Leung, Dodson, & Rowlands, 2017; Sørensen et al., 2012). Findings from this study support many aspects of Sørensen's health literacy model. The core aspects of knowledge, motivation, and competences are central to the development of health literacy capacities over time. Our findings show that self-efficacy plays an important role in the use of health literacy capacities. In the main, participants experienced increased confidence and self-efficacy in being able to manage their health. The focus of the cardiovascular risk reduction program contributed to an increased ability to access appropriate information as well as having a better understanding of that information. This is also linked to improvements in appraisal, as participants could question information and could discuss it with HCPs. The study findings have also highlighted the importance of both psychological and situational contexts that can impact on health literacy capacities, and this also supports the Sørensen framework. Although the model sets out empowerment as a possible outcome of health literacy, our findings suggest that empowerment as a process is also important. Another important finding, which is not addressed in the Sørensen model, is the role of the HCPs in the process of developing health literacy capacities.

Our study findings show that the HCP played a central role in both access and appraisal of information and could facilitate or impede gaining new knowledge (such as through the referral process). The relationship with, and perceived support from, the HCP were also important, and interactions with HCPs permeate all aspects of health literacy capacity development from accessing information through to support for use of health information. An important finding is the participants' reported willingness to remain with a GP even when there were concerns about the level of care and services provided. This is an important issue because GPs are generally the first point of access to health care services and play a key role in building patients' health literacy (Lausen et al., 2018). Health literacy capacity developments may be impeded when a person is reliant on a local health service provider they do not have confidence in or with whom they are not wholly comfortable.

At T1, many participants perceived having limited control/power across situations, which shifted toward having an increased sense of control as time went on. Although overall increases in confidence can be equated with self-efficacy and positive health literacy developments, developments can be stalled by the onset of illness, changed life circumstances, and/or poor communication experiences with HCPs. The broader context of the everyday life experiences of participants impacted on their abilities to positively use health literacy capacities and to sustain motivation and health practices over time. There is a greater need for HCPs to have increased insight into what is happening in people's lives and to be aware of particular vulnerable periods when additional supports may be required.

In particular, HCPs in the primary care setting could be more sensitive to recognizing when psychological supports might be needed and how they could be made available. HCPs are also well positioned to support patients to develop greater self-efficacy around their health-related activities as this contributes to enhancing motivation and empowerment. This is particularly relevant for patients with low health literacy (Lausen et al., 2018).

Overall, participants strived to manage their health and illness, particularly in relation to sustaining health practices, and identified the need to also manage psychological and emotional issues to be successful. This complements Lorig and Holman's (2003) definition of self-management, which includes the three components of medical, role, and emotional management. Findings also compare favorably with challenges to self-management put forward by Vallis (2009), which comprise barriers that are individually based (low skills, motivation, self-confidence, emotional distress), relationship based (e.g., lack of social support), and environmentally based (e.g., negative stimuli for healthy behavior in society).

Interactive and critical health literacy are acknowledged to play an important role in the successful management of chronic illness (Heijmans et al., 2015). The broader definition of critical health literacy put forward by Sykes, Wills, Rowlands, & Popple (2013) is relevant for the realities of daily management of health and illness highlighted in this study. This includes having the ability to appraise and analyze health information in a critical way and apply it to the context of their own lives (Sykes & Wills, 2018). This is evident in some of the issues raised in this study, such as the questioning of food-marketing practices and treatment and medication implications.

Participant experiences of interactions with the program staff lend support to preferences for access to community-based, holistic, and one-stop multidisciplinary service to assist in health and illness management. Promoting health literacy is a central strategy for improving self-management in health (Levin-Zamir & Peterburg, 2001). Building an explicit health literacy component into programs that focus on reduction of risk factors for various chronic illness as well as the improvement of secondary prevention is recommended for the delivery and evaluation of such programs (Magnani et al., 2018). This study highlighted individual variation in health literacy developments over time. It is crucial that HCPs are aware of the health literacy needs of service users to help foster positive developments in their health literacy. It would be particularly useful to focus on the development of critical health literacy competencies as advocated by Sykes and Wills (2018).

## Study Strengths and Limitations

A strength of the current study is the qualitative perspective, which allows for a more in-depth examination of the development of HL capacities from the perspective of study participants. The longitudinal qualitative study makes possible the identification of contextual and intervening conditions surrounding change (Saldana, 2002). The longitudinal aspect of this study also allowed us to identify the types of factors that can contribute to the positive development of health literacy for people over time.

The relatively small sample and the attrition of study participants from T1 to T3 is an important study limitation. It must be considered that those who left the study could have experienced additional barriers in terms of health literacy capacity development that are not accounted for in the study findings. However, after reviewing the data it is apparent that the experiences of the 17 study participants are sufficiently diverse in terms of health, illness, socio-demographic profile, and life experiences to provide a realistic account of experiences. This study focused on a specific population sample who attended a risk reduction program. It is possible that some of the positive effects in relation to the development of health literacy capacities are due in part to the effects of program participation. Ultimately, study participation relied on the voluntary participation of people, and so it is possible that those people who were most engaged with health issues were more likely to take part.

## Conclusion

Positive developments in health literacy capacities are important for the self-management of health and illness. Longitudinal findings underscore the importance of the HCP in supporting the development of health literacy capacities over time. These findings lend support to the need to integrate health literacy into medical and other HCP curricula to raise awareness of the concept of health literacy and to enhance HCPs communication strategies for patients with different health literacy skills (Kaper et al., 2018; Lausen et al., 2018).

## Figures and Tables

**Table 1:** Overview of Timeline, Sample, and Methods for Overall Longitudinal Qualitative Study

**Time Point**	**Number of Participants**	**Methods**
T1 (Baseline: beginning of program)	26	HLS-EU (2012) survey and interview completed
T2 (End of program at 12 weeks)	19	Interview completed
T3 (1-year follow up at 12 months)	17	HLS-EU (2012) survey and interview completed

Note. HLS-EU = European Health LIteracy Survey; T = time point.

**Table 2:** Profile of Study Participants (*N* = 17)

**Criteria**	***n* (%)**
Participants	10 (59)
Female	
Male	7 (41)

Age, *M* (range)	59 years (36-76 years)

Education (highest level attained)	
Primary school level (low)	2 (12)
Incomplete primary school (low)	1 (6)
Secondary-intermediate level (low)	7 (41)
Completed secondary (medium)	2 (12)
Diploma/certificate (medium)	2 (12)
Primary degree (high)	1 (6)
Postgraduate/higher degree (high)	2 (12)

Social class^a^	
I (high)	1 (6)
II (high)	5 (29)
III (medium)	0 (0)
IV (medium)	1 (6)
V (low)	2 (12)
VI (low)	2 (12)
VII (low)	6 (35)

General health literacy level from HLS-EU measure at T3	
Limited	5 (29)
Adequate	12 (71)

Health service access	
Private health insurance	8 (47)
Medical card only^b^	4 (23)
Private and medical card	3 (18)
Neither	2 (12)

Note.

a Central Statistic Office (2012).

b A medical card allows access to general practitioner services, community health services, dental services, prescription medicines, and hospital care free of charge under the General Medical Services Scheme for subgroups of the population based on income levels and/or specific medical conditions (Department of Public Expenditure and Reform, 2016; Health Service Executive, 2017). HLS-EU = European Health Literacy Survey; T = time point.

**Table 3:** Validation Criteria

**Criteria**	**Description**
Credibility	Participants' perspectives were reported as accurately as possible and the participants' own voices are used. Review and refinement of themes through a consensus process was undertaken among the three authors
Triangulation	Convergence was sought among multiple sources of information (interview transcripts, memos, relevant theory, and researchers' analysis) to verify interview data and to develop themes. A level of member checking was achieved where key issues and themes arising at T1 were reviewed with the participants at the start of T2 and T3 interviews
Transferability	Detailed accounts of the data and the context of data collection are provided
Descriptive validity	Multiple reading of the transcripts took place and recordings were listened to in line with the methodology of thematic analysis (Braun & Clarke, 2006)
Interpretive validity	The study participants' voices were relied on as much as possible for interpretation of meaning alongside the meaning attributed by the researcher
Theoretical validity	The findings were clearly set out within relevant theory in the field of health literacy
Researcher reflexivity	Preliminary analysis between time points allowed the researchers to reflect on personal assumptions related to health literacy and social contexts

Note. T = time point.

**Table 4:** Themes Together with Subthemes and Categories

**Theme**	**Subtheme**	**Categories**
Gaining control and becoming empowered	Psychological impacts of external events	Dealing with stress Dealing with pain Past negative health care experiences/delayed diagnosis
	Self-efficacy	Ability and confidence to make lifestyle changes
	Looking after self	Need for psychological supports Fears and anxieties Dealing with grief
Embedding knowledge, health practices, and motivation	Accessing and using information	Awareness of limitations Knowledge on diet and exercise
	Environment	Urban/rural Facilitator/barrier to health promotion practices
	Food literacy (what shapes food choices)	Marketing Food labels Family/social aspects
Dynamics of relationship and support of health care providers	Communication, rapport, trust, and approachability	Positive/negative outcomes Non-paternalistic
Treatment decision-making	Managing and challenging side effects Decisions about treatment	Fears and misconceptions Logistical and practical considerations

**Figure A.**
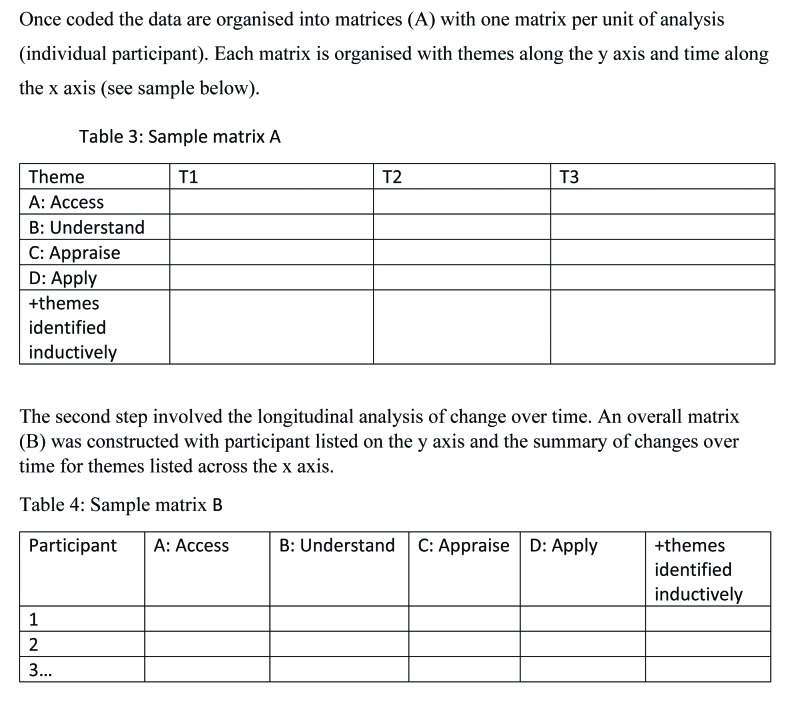
Trajectory analysis. Adapted from “Analyzing longitudinal qualitative data: The application of trajectory and recurrent cross-sectional approaches,” by D. Grossoehme, and E. Lipstein, 2016, *BMC Research Notes, 9*, p. 136.

**Table A:** Longitudinal Question Frameworks

**Saldana (2002, 2003)**	**Lewis (2007)**
Framing questions (situate the context of the data) What is different from one round of data to the next (difference in confidence, perceived sense of control)? When do changes occur through time (what is the timing of changes)? What contextual and intervening conditions appear to influence and affect participant change through time (what external events are happening; what illness experience occurs)? What are the dynamics of participant changes through time (make comparisons between participants in relation to changes in health literacy levels, changes, and illness experiences. When were the effects of intervening factors on changes and comparisons of these across participants)? What preliminary assertions abut participant changes can be made as data analysis progresses (empowerment across time points becoming apparent)? Descriptive questions What increases/emerges through time (need to focus on self)? What is cumulative through time (confidence and self efficacy)? What kind of surges/epiphanies occur through time (role of health care provider)? What decreases/ceases through time (fears and anxieties abated for many)? What remains constant or consistent through time (engaging with health information; reluctance to change providers)? What is idiosyncratic through time (is health literacy development orderly or consistent? How does health literacy play out in different circumstances?) What is missing through time (limited changes for some)? Analytic and interpretive questions What changes interrelate through time (illness, adverse life experiences and confidence, sense of control)? What changes through time oppose or harmonize with natural human development or constructed social processes (expected changes at time point 2 but were generally sustained over following 9-month period)? What are participant or conceptual rhythms such as cycles through time (participants dealt with new diagnosis, changing symptoms, and ongoing management)? What is the through-line of the study (perceived control is central to health literacy development)?	Descriptive question What is the type, extent, and timing of any changes (linked to framing questions above)? Location question Who showed changes, when, and in what contexts (linked to framing questions above)? Explanation question (drivers for change) What were the factors that influenced the changes? (changes in confidence; external event)? Evaluation question What influenced the experience of participants in use of health literacy capacities and change/lack of change? (relationship with health care provider; being able to use information; confidence)? Consequences question What was the effect of further changes, new directions, loss of opportunity (more positive outlooks, enhanced health care provider interactions, increased interactive and critical health literacy apparent)? Personal meaning question What was the perceived importance of the change (very positive)? Policy meanings Applied to Sørensen framework (Sørensen et al., 2012) Researcher framework Reflexivity
